# Localization of Multi-Lamellar Vesicle Nanoparticles to Injured Brain Tissue in a Controlled Cortical Impact Injury Model of Traumatic Brain Injury in Rodents

**DOI:** 10.1089/neur.2021.0049

**Published:** 2022-04-05

**Authors:** Ricky Whitener, Jeremy J. Henchir, Thomas A. Miller, Emily Levy, Aubrienne Krysiewicz-Bell, Eliza S. LaRovere Abrams, Shaun W. Carlson, Naresh Menon, C. Edward Dixon, Michael J. Whalen, Claude J. Rogers

**Affiliations:** ^1^ ChromoLogic LLC, Monrovia, California, USA.; ^2^ Neurological Surgery, University of Pittsburgh, Pittsburgh, Pennsylvania, USA.; ^3^ VA Pittsburgh Healthcare System, Pittsburgh, Pennsylvania, USA.; ^4^ Department of Pediatrics/Critical Care Medicine, Massachusetts General Hospital and Harvard Medical School, Boston, Massachusetts, USA.

**Keywords:** blood–brain barrier, controlled cortical impact, *in vivo* studies, traumatic brain injury

## Abstract

Severe traumatic brain injury (TBI), such as that suffered by patients with cerebral contusion, is a major cause of death and disability in young persons. Effective therapeutics to treat or mitigate the effects of severe TBI are lacking, in part because drug delivery to the injured brain remains a challenge. Promising therapeutics targeting secondary injury mechanisms may have poor pharmacokinetics/pharmacodynamics, unwanted side effects, or high hydrophobicity. To address these challenges, we have developed a multi-lamellar vesicle nanoparticle (MLV-NP) formulation with a narrow size distribution (243 nm in diameter, 0.09 polydispersity index) and the capability of encapsulating hydrophobic small molecule drugs for delivery to the injured brain. To demonstrate the utility of these particles, we produced dual-fluorescent labeled nanoparticles containing the organic dyes, coumarin 153 and rhodamine B, that were delivered intravenously to Sprague-Dawley rats and C57Bl6/J mice at 1, 1 and 4, 24, or 48 h after controlled cortical impact injury. Distribution of particles was measured at 5, 25, 48, or 49 h post-injury by fluorescence microscopy of coronal brain sections. In all cases of MLV administration, a 1.2- to 1.9-fold enhancement of ipsilateral fluorescence signal was observed compared to the contralateral cortex. Enhanced fluorescence was also observed in the injured hippocampal tissue in these animals. MLV-NPs administered at 1 h were observed intracellularly in the injured hemisphere at 48 h, suggesting the possibility of concentrated drug delivery to injured cells. These results suggest that MLV-NP delivery of therapeutic agents may be a viable strategy for treating cerebral contusion TBI.

## Introduction

Traumatic brain injury (TBI) is a major cause of death and disability in the United States, resulting in 2.87 million deaths, hospitalizations, and emergency room visits annually.^1^ Brain injuries affect the physical and emotional health of patients and their families,^2,3^ with an estimated economic loss of $76.5 billion annually.^4,5^ These injuries may lead to irreversible functional loss from tissue damage and disruption of neural circuits for cognitive and sensorimotor functions.^6,7^ TBI results in physiological changes attributable to calcium release, accumulation of reactive nitrogen and oxygen species, mitochondrial dysfunction, glutamate toxicity, and neuroinflammation, leading to chronic progressive neurodegeneration.^8–12^ Further, a positive feedback loop is generated from the injury, starting with these biochemical derangements plus damage-associated molecular patterns (DAMPs). This leads to further cell death and continuing release of biochemical derangements and DAMPs, perpetuating secondary injury.^13,14^ This complex pathology limits available therapeutic options.^15^

Under normal physiological conditions, the blood–brain barrier (BBB) is an obstacle for systemic delivery of therapeutics to the brain.^16^ However, TBI damages the BBB, which may result in acute permeability.^17,18^ The “leaky” BBB offers the possibility for nanoparticle-based drug delivery directly to injured regions of the brain.^19^ Nanoparticles (NPs) are solid colloidal particles ranging from 1 to 1000 nm in size.^20–22^ Their size, mobility, and pharmacological attributes can be utilized for various biomedical applications. Engineered NPs can be programmed to have unique functional and structural organization,^23,24^ which can allow for targeted delivery of hydrophobic and -philic drugs. Over two dozen NP platforms have been approved by the U.S. Food and Drug Administration for clinical use to either treat or diagnose diseases, with many more currently in clinical trials.^25,26^ Further, NP delivery systems can have their *in vivo* residence times altered by manipulating key parameters, such as size, charge, shape, and surface coating molecules.^27^

Liposomes have been extensively tested and used for drug delivery to the brain.^28^ Because of their composition, multi-lamellar vesicle (MLV) NPs made from phospholipid bilayers provide a unique opportunity to deliver therapeutics into cells. MLV-NPs have ideal properties for drug delivery to the brain because they are non-toxic, biocompatible, biodegradable, able to cross the BBB, and maintain physical stability in blood with prolonged blood circulation.^29,30^ The addition of polyethylene glycol (PEG) to the surface of MLV-NPs reduces clearance, improves biocompatibility and stability, and prevents aggregation in the blood.^31–34^ PEG can provide a partial negative charge to NPs, and neutral to moderately negatively charged NPs have been demonstrated to show no toxicity when utilized for brain delivery.^35^ The size and shape of NPs can affect the uptake pathways and localization in the brain.^36,37^ Finally, surface modifications can include targeting moieties capable of localizing particles to sites of injury, for example, targeting based on changes in cell-surface protein expressions from impact of injury.

We have engineered PEGylated MLV-NPs with an average diameter of 250 nm that contain two fluorescent dyes, coumarin 153 (C153) and rhodamine B (RhoB), for visualization (C153-RhoB-MLVs). These particles were used to monitor localization in the brain post-TBI, induced in rats and mice using the controlled cortical impact (CCI) model,^38^ to determine the viability of MLV-NPs to deliver neuroprotective therapeutics. Here, we administered C153-RhoB-MLV NPs intravenously (i.v.) to rats and mice at different time points post-CCI (1, 24, and 48 h) and examined their distribution in ipsi- and contralateral brain regions after allowing the particles to circulate in the animals for 1, 4, or 48 h after administration. These MLV-NPs are based on a liposomal formulation that is able to reach and penetrate the BBB and, potentially, release neuroprotective therapeutics in a sustained manner. Therefore, effective localization of these particles suggests that MLV-NPs may be an effective drug-delivery vehicle to treat secondary injury processes after contusion TBI.

## Methods

### Synthesis of coumarin 153/rhodamine B/multi-lamellar vesicle nanoparticles

MLVs were generated using a liposomal formulation technique with improved drug bioavailability and particle stability. MLVs were formed through covalently crosslinking functionalized head groups of adjacent lipid bilayers. This method is an established multi-step procedure based on conventional dehydration-rehydration methods^39,40^ by incorporation of a thiol-reactive maleimide head-group lipid, covalently binding through the use of dithiothreitol. The last step included PEGylation of the MLV surface.

### Characterization of coumarin 153/rhodamine B/multi-lamellar vesicle nanoparticles

Transmission electron microscopy (TEM) characterization of C153-RhoB-MLV NPs was performed by applying a 200-MESH carbon-coated grid to the glow discharge chamber, then adding the C153-RhoB-MLV NPs to the carbon-coated grid. Then, 1% uranyl acetate solution was added, followed by deionized water. The liquid was removed by applying filter paper to the edge of the grid, and this wash step was repeated two more times. Magnification of the image was adjusted for particles in the size range of 250 nm before micrographs were recorded.

Dynamic light scattering (DLS; ZetaPALS; Brookhaven Instruments, Holtsville, NY) was used to measure size distribution of MLV-NPs based on the average of 10 reads, with values ranging from 300 to 500 × 1000 counts per s^−1^. Particles where 95% of the sample had a diameter within 88–312 nm and a polydispersity index (PDI) <0.7 were used for subsequent studies.

### Toxicity assay

U-251 MG cells were treated with C153-RhoB-MLV NPs in 20 μL of Dulbecco's (DPBS) phosphate-buffered saline (PBS), at concentrations ranging from 0 to 10^3^ μg/mL. After incubating for 1 h at 37°C, cells were spun at 100*g*, the supernatant was removed, and cells were treated with trypsin. Cells were then spun down at 350*g* for 5 min, and the supernatant was removed. Cells were resuspended in DPBS. An aliquot was removed, and toxicity was determined using the XTT Cell Viability Kit (Biotium, Inc., Fremont, CA).

### In vitro *localization*

Human brain cerebral microvascular endothelial cells (HBMECs) were treated with 5 μL of CellBrite™ (CB) red membrane dye, then washed three times with medium, and resuspended. Next, 7.2 × 10^4^ cells were plated in 200 μL of media, and 50 μL of 10^3^ μg/mL C153-MLV NPs were added to the culture. After incubating for 3 h, media was removed and cells were rinsed, washed twice with PBS, and fixed with formaldehyde. Cells were washed with PBS and imaged by fluorescent microscopy.

### In vivo *rat localization and controlled cortical impact*

Adult male Sprague-Dawley rats (Harlan Laboratories, Indianapolis, IN) with a body weight of 325–375 g were anesthetized with 4% isoflurane and a 2:1 N_2_O/O_2_ mixture in a vented anesthesia chamber. After endotracheal intubation, rats were mechanically ventilated with a 2% isoflurane mixture.

An indwelling right jugular i.v. catheter was placed before TBI. Under the same anesthesia, a catheter was placed and secured in the right jugular vein. The catheter was tunneled under the skin and secured between the clavicles to prevent dislodgement.

After jugular catheter implantation, rats were mounted in a stereotaxic frame in a prone position secured by ear bars and an incisor bar. The topical analgesia, EMLA™ cream (lidocaine and prilocaine 2.5%), was applied to the tips of the ear bars. The head was held in a horizontal plane with respect to the interaural line, shaved, and swabbed with betadine. Using aseptic conditions, a midline incision was made, soft tissues reflected, and a craniectomy was made. The injury was produced by a 6-mm-diameter impactor tip deforming the tissue for 2.6 mm at a velocity of 4 m/s. Cerebral contusion was induced over the right parietal area (center of craniectomy at anteroposterior, +4.0 mm; lateral, +2.8 mm from lambda). After CCI, the skin over the craniotomy site was sutured and the animal was extubated. EMLA was used to alleviate acute post-surgery wound pain. Sham animals underwent all surgical procedures, but did not receive a cortical impact. During the surgery, core body temperature was monitored continuously by a rectal thermistor probe and maintained at 37°C–38°C.

C153-RhoB-MLV particles (4.6 mg/mL) were administered through indwelling jugular cannulas (150-μL injection in PBS per dose) at 1 and 4 h after injury for a total dose of 9.2 mg/kg and euthanized 1 h after the last injection. At the end of each experiment, animals were transcardially perfused with cold saline followed by 10% neutral buffered formalin. Brains and organs were removed and stored in the fixative for 48 h and then were transferred to phosphate-buffered sucrose solution for cryoprotection. Microtome-cut (35 μm) sections were imaged and regional fluorescein staining in brain, kidney, and liver slices was quantified to measure NP accumulation.

### In vivo *mice localization and controlled cortical impact*

Anesthesia was induced in C57Bl6/J mice (Jackson Labs, Bar Harbor, ME) using a Fluotec 3 vaporizer (Colonial Medical, Amherst, NH) and 70% nitrous oxide, 4–5% isoflurane (Anaquest, Memphis, TN), and balance oxygen. After induction, mice were positioned on a stereotaxic frame with the nose placed in an opening of a plastic tubing carrying anesthesia from the chamber to the animal and then out into a negative pressure hood. Isoflurane was reduced to 3% and mice tested for adequate depth of anesthesia by tail pinch. A 5-mm craniotomy was made with a portable drill and trephine over the left parietotemporal cortex. The bone flap was removed with the dura intact. Impact was delivered using a 3-mm flat-tip pneumatic piston at a velocity of 6 m/s, duration 100 ms, and depth of 0.6 mm. The bone flap was discarded and the scalp sutured closed.

C153-RhoB-MLV NPs (0.667 mg/mL) were administered by tail vein injection (300 μL in PBS, 10 mg/kg) at 1, 4, 24, or 48 h after injury in mice that were briefly anesthetized with isoflurane. At 1 or 4 h post-injection, mice were transcardially perfused with 100 mL of PBS. Brains were removed and fixed for 24 h in 4% paraformaldehyde, then cryoprotected in 30% sucrose solution for another 24 h. Brains were frozen at −80°C and sectioned on a cryostat (20 μm) through the contusion, taking sections every 300 μm. Brain sections were mounted on glass slides, cover-slipped with PBS, and imaged to measure particle localization and accumulation.

### Microscopy and analysis of particle fluorescence

Rat tissue sections were imaged at 10 × magnification on a C2 Nikon 90i microscope (Nikon, Melville, NY), using consistent laser and camera settings. Mouse tissue sections were imaged on a Nikon Ti300 fluorescence microscope at 20 × magnification. For all animals, mean gray density of green fluorescence in ipsi- and contralateral micrographs of cortical sections was obtained using ImageJ software (National Institutes of Health, Bethesda, MD), and values were averaged for each animal.

### Statistical analysis

A ratio of ipsi- to contralateral cortex was obtained and plotted for each animal at each time point. The paired Student's *t*-test was used to compare mean gray values in ipsi- versus contralateral images for each animal per group. A minimum sample size of *N* = 6 was chosen based on power analysis for the paired *t*-test given an experimental power of 80%, assuming a 70% difference in means.

## Results

**FIG. 1.**
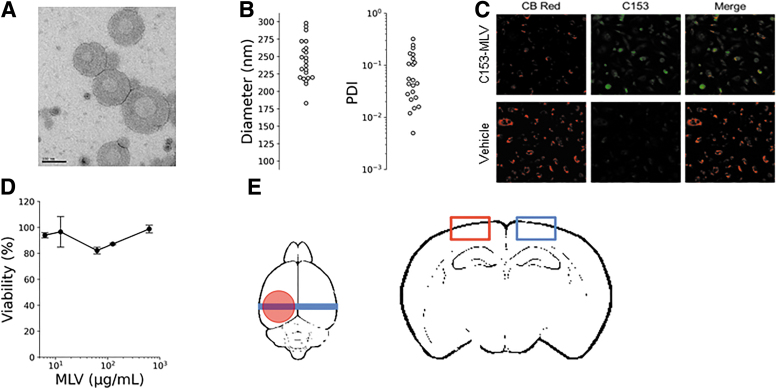
Characterization of MLV-NPs. (**A**) Representative TEM image of the MLV nanoparticles showing the multi-lamellar structure. An inner compartment is visible, and distinct particles have formed at the expected size range. (**B**) DLS size and PDI values for 22 independent preparations of MLV-NP. The particles have an average size of 243 ± 30 nm and a PDI of 0.09. (**C**) C153-MLVs localize on the surface of human brain microvascular endothelial cells (HBMECs). PBS control proves fluorescence is generated from C153. (**D**) Cell viability in the presence of C153-MLVs. No reduction in U-251 MG cell viability was observed for cells exposed to C153-MLVs after 72 h. (**E**) Overview of MLV-NP localization studies showing the approximate location of the CCI (red circle), coronal sections (blue bar), and ipsilateral and contralateral ROIs (red and blue rectangles, respectively) within a coronal tissue section. C153, coumarin 153; CCI, controlled cortical impact; DLS, dynamic light scattering; MLV-NPs, multi-lamellar vesicle nanoparticles; PBS, phosphate-buffering saline; PDI, polydispersity index; TEM, transmission electron microscopy.

The multi-lamellar structure of the NPs was validated by TEM (Fig. 1A). Particle size and polydispersity were measured using DLS, and homogenous size distributions of ∼250 ± 25 nm were confirmed for all particles used for *in vivo* studies (Fig. 1B). In order to visualize the C153-RhoB-MLVs in tissue sections, the organic dyes, C153 and RhoB, were selected because of their charge, size, and hydrophobic properties. For this study, particles were loaded with 75 μg/mL of C153 and RhoB. Particles localized to HBMECs *in vitro* (Fig. 1C). HBMECs were treated with C153-MLV particles and CB Red membrane dye and visualized using confocal microscopy to confirm that MLV particles could be visualized near the surface of HBMECs. Further, particles were confirmed to be non-cytotoxic using the XTT assay over a MLV particle concentration range spanning 3 orders of magnitude in U-251 MG cells (Fig. 1D).

### Localization of coumarin 153/rhodamine B/multi-lamellar vesicle particles in the brain after a controlled cortical impact injury in rats and mice

**FIG. 2.**
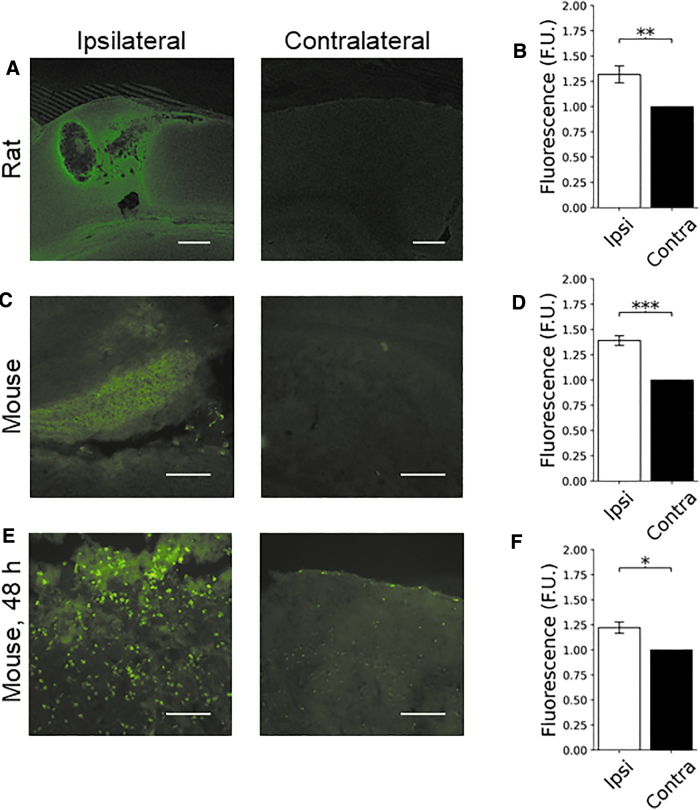
MLV-NP localization in rat and mouse CCI models. C153-RhoB-MLV particles were given to rodents at 1 and 4 h, then examined at 5 h post-injury. Increased ipsilateral fluorescence was observed in both (**A**) rats (*N* = 10) and (**C**) mice (*N* = 8). (**B**) Quantification of the signal resulted in a 1.32 ± 0.08-fold enhancement in rats (*p* = 0.004). (**D**) Similarly, a 1.39 ± 0.05-fold enhancement of ipsilateral fluorescence was observed in mice (*p* = 0.00008). (**E**) C153-RhoB-MLV particles were injected 1 h post-injury and examined 48 h (*N* = 6) post-CCI. (**F**) Enhancement of1.22 ± 0.06-fold was observed at 48 h (*p* = 0.011). Levels of enhancement were consistent with those observed after 5 h. (E) At 48 h, fluorescent puncta were observed in the ipsilateral cortex, suggesting cellular uptake of particles or dye. Scale bar = 100 μm. C153, coumarin 153; CCI, controlled cortical impact; MLV-NPs, multi-lamellar vesicle nanoparticles; RhoB, rhodamine B.

C153-RhoB-MLVs were injected at 1 and 4 h post-CCI, and brains were examined at 5 h post-injury. Coronal sections (Fig. 1E) of the brain showed increased localization of C153-RhoB-MLVs to the injured ipsilateral cortex, compared to the uninjured contralateral cortex, in both rats (1.32 ± 0.08-fold [*N* = 10], *p* = 0.004; Fig. 2A,B and Table 1) and mice (1.39 ± 0.05-fold [*N* = 8], *p* = 8.4 × 10^−5^; Fig. 2C,D and Table 1). This increase was not attributable to the injury, given that baseline autofluorescence decreased in the ipsilateral cortex compared to the contralateral cortex after CCI injury in mice administered with vehicle (PBS) alone (0.89 ± 0.4-fold [*N* = 6], *p* = 0.041; Supplementary Fig. S1 and Table 1). No obvious accumulation of fluorescent signal was observed in the kidney or liver in rats (Supplementary Fig. S2).

**Table 1.** Summary of MLV Localization in the Injured Rodent Cortex Post-CCI

Species	Reagent	Administration (post-CCI)	Euthanized (post-CCI)	*N*	Ipsilateral fluorescence fold-change	*p* value
Rat	C153-RhoB-MLV	1 and 4 h	5 h	10	1.32 ± 0.08	0.004
Mouse	C153-RhoB-MLV	1 and 4 h	5 h	8	1.39 ± 0.05	8.4 × 10^−5^
Mouse	C153-RhoB-MLV	1 h	48 h	6	1.22 ± 0.06	0.011
Mouse	PBS	1 h	5 h	6	0.89 ± 0.40	0.041
Mouse	C153-RhoB-MLV	24 h	25 h	6	1.54 ± 0.07	0.0008
Mouse	C253-Rho-MLV	48 h	49 h	7	1.88 ± 0.12	0.0020

Rodents were administered C153-RhoB-MLV nanoparticles in PBS or PBS alone at various times post-CCI and then euthanized 1–47 h later. The fold-change in ipsi- versus contralateral fluorescence in cortical tissue was quantified.

MLV, multi-lamellar vesicle; CCI, controlled cortical impact; C153, coumarin 153; RhoB, rhodamine B; PBS, phosphate-buffered saline.

### The apparent enhancement in ipsilateral fluorescence attributable to coumarin 153/rhodamine B/multi-lamellar vesicle localization is maintained for at least 48 h

Mice administered MLV-NPs at 1 h post-CCI and examined 48 h post-CCI showed a 1.22 ± 0.06-fold (*N* = 6, *p* = 0.011) enhancement of fluorescent signal in the ipsilateral cortex versus the contralateral cortex (Fig. 2E,F; Table 1). The presence of fluorescent puncta suggested cellular uptake of particles or dye (Fig. 2E; Table 1).

**FIG. 3.**
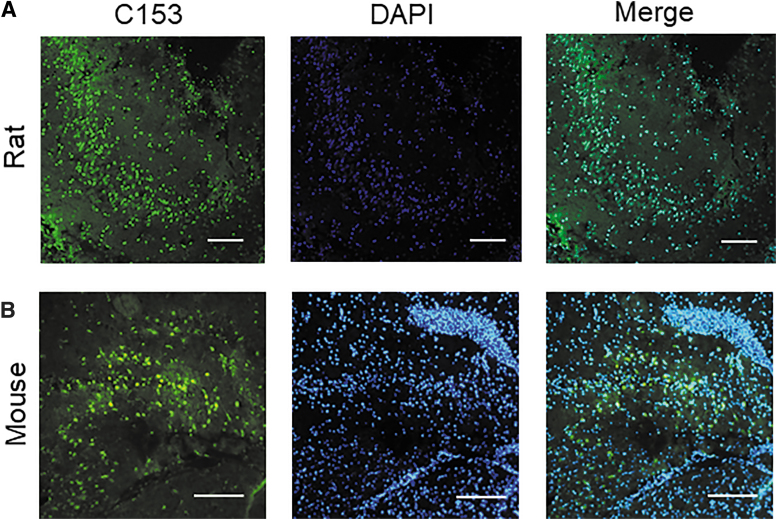
MLV-NPs localize in the injured hippocampal tissue in rat and mouse CCI models. (**A**) C153-RhoB-MLV particles were injected 1 and 4 h post-CCI in rats (*N* = 2) and examined at 5 h. (**B**) Similarly, particles were administered at 1 h post-CCI in mice, and tissue sections of the hippocampus were examined 48 h post-injury in mice (*N* = 4). Presence of bright fluorescent puncta suggests possible cellular uptake of particles, possibly by cells with compromised plasma membranes, or transfer of the dye. Scale bar = 100 μm. C153, coumarin 153; CCI, controlled cortical impact; MLV-NPs, multi-lamellar vesicle nanoparticles; RhoB, rhodamine B.

In addition to the cortex, MLV-NP signal was observed in the injured hippocampus after acute administration (1 and 4 h in rats, 1 h in mice; Fig. 3). Bright fluorescent puncta showed a strong signal indicating particle presence in both rats and mice, similar to the signal observed in mouse cortex with MLV-NPs delivered after 48 h. Overlap with nuclear staining (4′,6-diamidino-2-phenylindole; DAPI) confirmed either cellular uptake of particles or transfer of the dye to cells.

### Localization of coumarin 153/rhodamine B/multi-lamellar vesicle particles in the brain administered 24 or 48 h after controlled cortical impact injury

**FIG. 4.**
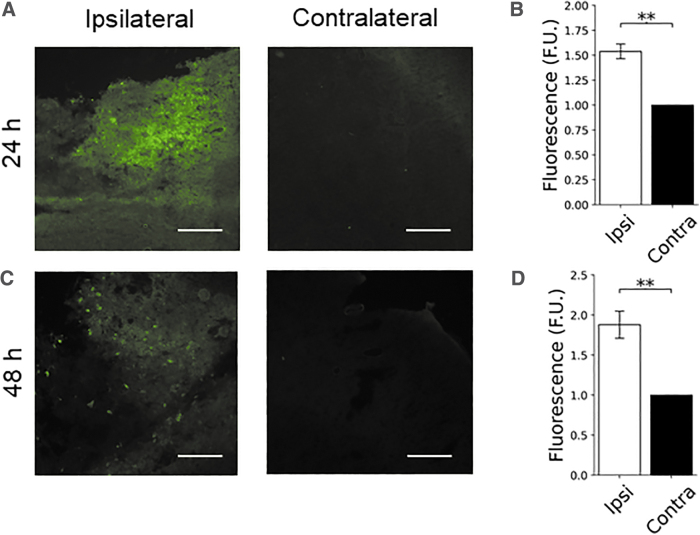
MLV-NPs localize to injured brain at least 48 h post-CCI injury in mice. C153-RhoB-MLV particles were injected (**A**) 24 h (*N* = 6) or (**C**) 48 h (*N* = 7) post-CCI and examined 1 h later. (**B**) Enhancement of 1.54 ± 0.07-fold of ipsilateral fluorescent signal was observed at 25 h (*p* = 0.0008), and (**D**) enhancement of 1.88 ± 0.12-fold was observed at 49 h (*p* = 0.0020). Levels of enhancement were consistent with those observed after 5 h (Fig. 2). Scale bar = 100 μm. C153, coumarin 153; CCI, controlled cortical impact; MLV-NPs, multi-lamellar vesicle nanoparticles; RhoB, rhodamine B.

Next, we tested whether particles could still be detected in the injured brain beyond the first 4 h post-injury. Mice were administered C153-RhoB-MLV particles 24 or 48 h post-CCI in a single injection, and brains of the animals were examined 1 h later. Consistent with the acute administration experiments, significant enhancement was observed in the ipsilateral cortex versus contralateral cortex in animals administered MLVs at 24 h (1.54 ± 0.07-fold [*N* = 6], *p* = 0.0008) and 48 h (1.88 ± 0.12-fold [*N* = 7], *p* = 0.0020; Fig. 4 and Table 1).

## Discussion

Our results suggest that MLV-NPs can be formulated to encapsulate hydrophobic small molecules, such as drugs and dyes, and maintain narrow size distributions (Fig. 1B) for *in vivo* drug delivery. The particles are non-cytotoxic (Fig. 1E) and lyophilizable, which improves their utility and offers the possibility for improved shelf-life compared to non-lyophilizable formulations.

An important strength of the CCI model is its scalability between species.^38^ When scaling injury parameters across species, a recommended starting point is to normalize the percent of brain volume deformed relative to the total brain volume.^38^ In the present study, the injury severity produced by CCI was comparable between the two species, as indicated by reports of similar losses of ipsilateral hemispheric tissue.^41,42^

Administration of particles to rodents after CCI injury resulted in localization of the particles to the injured region of the brain at all time points tested. Acute administration (1 and 4 h post-CCI) resulted in increased fluorescence signal in the ipsilateral cortex at 5 h in both species (Fig. 2). Similar levels of ipsilateral-specific enhancement of signal (1.2-fold) were observed 48 h after administration of particles at 1 h post-CCI in mice (Fig. 2E,F), suggesting that these particles could be used for long-term release of drugs. These results could be explained by BBB permeability to MLVs^43^ or adhesion to the endothelium in the damaged brain regions, as suggested by *in vitro* experiments. Thus, the combined effects of increased BBB permeability and reduced cerebral blood flow in injured hemispheres after CCI in mice and rats^44,45^ may result in an enhanced permeability and retention effect that localizes NPs. Previous studies have shown that NPs localize and accumulate near the site of injury after acute administration post-TBI.^46,47^

To our knowledge, this the first study to show that MLV NPs are able to deliver a payload of hydrophobic compounds to injured brain regions in a cerebral contusion model. MLV nanoparticles are fundamentally different from other types of nanoparticles that have been shown to localize to the injured brain in terms of size, composition, payload type, release rate, clearance rates, etc. The key differences to note are that MLVs, especially the PEGylated MLVs used in this study, have been shown to have prolonged circulating half-life, reduced macrophage uptake, and good drug release rates *in vivo*.^40,48,49^ Because of these properties, we believe the findings presented in our article are noteworthy because they demonstrate the potential for the drug delivery of classes of drugs that are not well suited for the other NP types demonstrated to localize to the injured brain.

For clinical applications, the need to administer drugs within the first few hours post-injury would limit the potential of these MLVs as a drug-delivery vehicle. Mice administered MLVs 24 or 48 h after injury showed similar levels of ipsilateral fluorescence enhancement (1.5- to 1.9-fold; Fig. 4) to animals administered particles acutely. This suggests that the particles are still able to cross the BBB at least 48 h after injury. If similar dynamics occur clinically in TBI patients, these results suggest that drugs delivered with this MLV formulation may have a reasonably high therapeutic window.

When examined 5 h post-injury, the signal in the ipsilateral cortex appeared brighter near lesions, but was otherwise diffuse. After 48 h, MLVs administered at 1 h appeared to localize intracellularly, given the bright puncta that overlayed with DAPI signal (Fig. 2E). This may be attributable to active cellular uptake or could be attributable to entry of the particles into degenerative cells with plasmalemma damage. In animals given MLV NPs 48 h post-injury and examined 1 h later, fewer puncta were observed, suggesting that internalization into cells takes longer than 1 h (Fig. 4C). However, MLV localization in the hippocampus was also characterized by the presence of puncta in rats administered MLVs at 1 and 4 h post-injury and examined at 5 h (Fig. 3A). In mice, similar localization in hippocampal tissue also occurred (Fig. 3B), consistent with the behavior in the cortex for that species.

Most studies were performed exclusively in mice because of the reduced quantity of MLVs required in the smaller animals. However, the similarity in both fold-enhancement in injured tissue and MLV detection in both the cortex and hippocampus in two rodent species strengthens the confidence in the results of the studies. Further, injury appeared to reduce autofluorescence in the ipsilateral cortex compared to the contralateral cortex (Supplementary Fig. S1). This suggests that the increase in concentration of MLVs in the injured tissue may be higher than these measurements suggest, and that labeling in the injured brain regions with MLVs is not an artifact of increased brain tissue autofluorescence.

## Conclusion

Localization of fluorescently labeled MLV-NPs was specific to injured brain tissues in rodents that received CCI. The particles remained in the injured cortex for at least 48 h and could be visualized in the injured brain at least 48 h after injury. The NPs are easy to produce, can encapsulate hydrophobic organic molecules, are non-cytotoxic, and can be lyophilized and reconstituted for use. This suggests that these particles may be an effective drug-delivery vehicle to mitigate neuronal damage after TBI.

## Supplementary Material

Supplemental data

Supplemental data

## Abbreviations Used

BBB: blood–brain barrier
C153: coumarin 153
CB: CellBrite
CCI: controlled cortical impact
DAMPs: damage-associated molecular patterns
DAPI: 4′,6-diamidino-2-phenylindole
DLS: dynamic light scattering
DPBS: Dulbecco's phosphate-buffered saline
HBMECs: human brain cerebral microvascular endothelial cells
i.v.: intravenous(ly)
MLV: multi-lamellar vesicle
NPs: nanoparticles
PBS: phosphate-buffered saline
PDI: polydispersity index
PEG: polyethylene glycol
RhoB: rhodamine B
TBI: traumatic brain injury
TEM: transmission electron microscopy
