# Supplementary material for: Localization of Multi-Lamellar Vesicle Nanoparticles to Injured Brain Tissue in a Controlled Cortical Impact Injury Model of Traumatic Brain Injury in Rodents
Source: Neurotrauma Rep. 2022 Apr 5;3(1):158–67. doi: 10.1089/neur.2021.0049 (PMC8985535; doi:10.1089/neur.2021.0049)
Supplement: Supplemental data [file Suppl_FigureS1.pptx]

## Slide 1
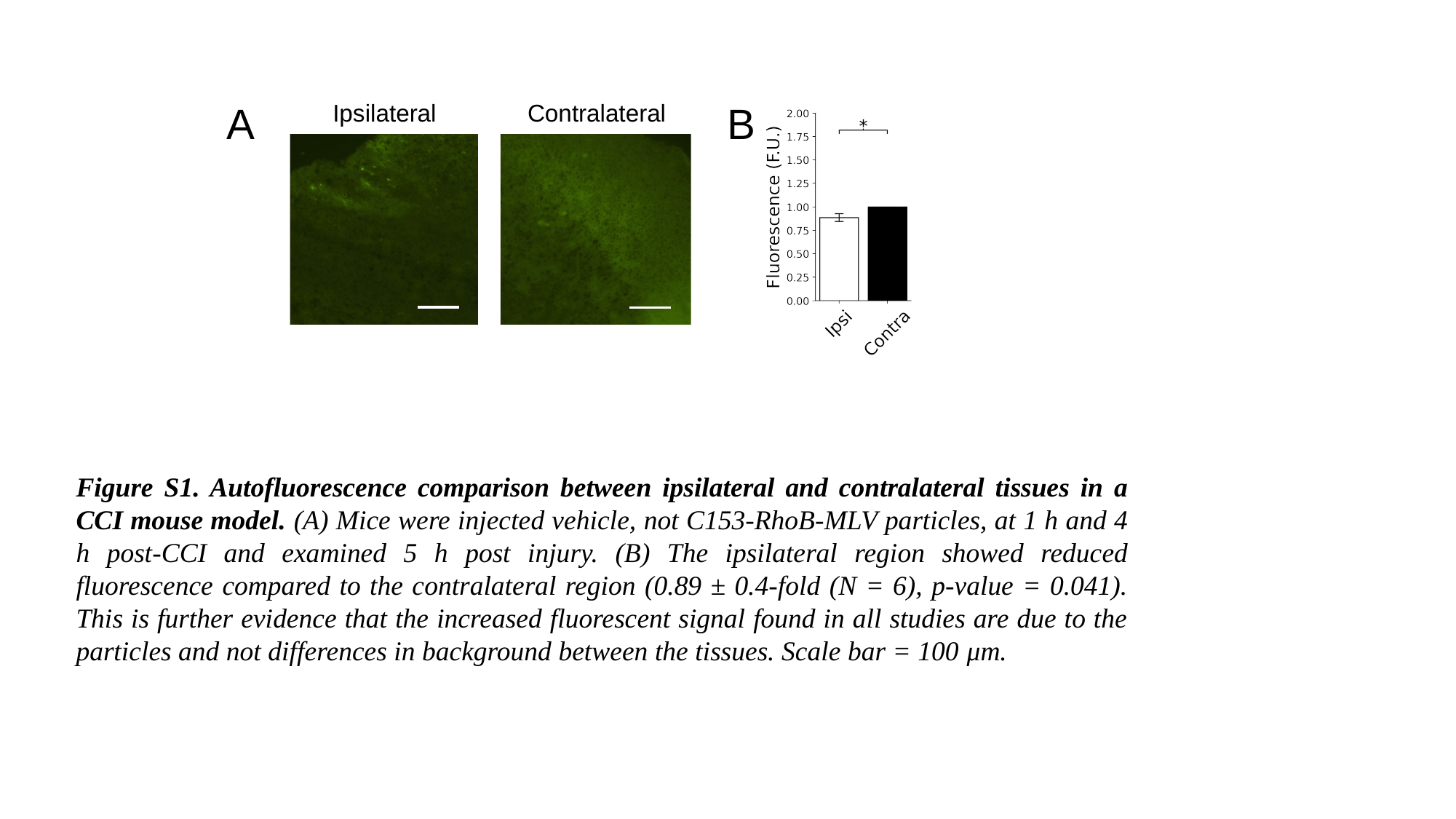

A
B
Contralateral
Ipsilateral
Figure S1. Autofluorescence comparison between ipsilateral and contralateral tissues in a CCI mouse model. (A) Mice were injected vehicle, not C153-RhoB-MLV particles, at 1 h and 4 h post-CCI and examined 5 h post injury. (B) The ipsilateral region showed reduced fluorescence compared to the contralateral region (0.89 ± 0.4-fold (N = 6), p-value = 0.041). This is further evidence that the increased fluorescent signal found in all studies are due to the particles and not differences in background between the tissues. Scale bar = 100 μm.
