# Supplementary material for: Localization of Multi-Lamellar Vesicle Nanoparticles to Injured Brain Tissue in a Controlled Cortical Impact Injury Model of Traumatic Brain Injury in Rodents
Source: Neurotrauma Rep. 2022 Apr 5;3(1):158–67. doi: 10.1089/neur.2021.0049 (PMC8985535; doi:10.1089/neur.2021.0049)
Supplement: Supplemental data [file Suppl_FigureS2.pptx]

## Slide 1
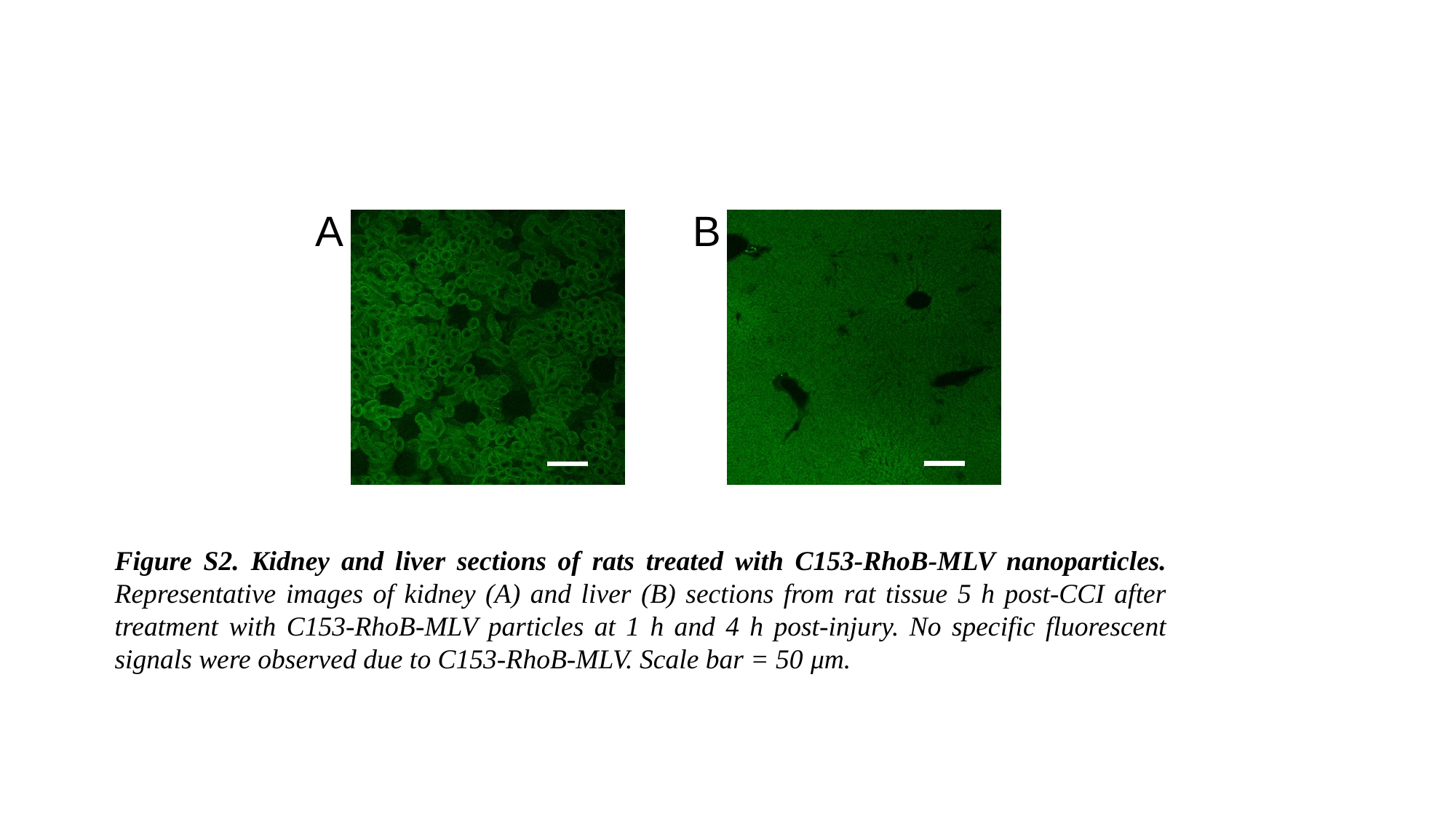

A
B
Figure S2. Kidney and liver sections of rats treated with C153-RhoB-MLV nanoparticles. Representative images of kidney (A) and liver (B) sections from rat tissue 5 h post-CCI after treatment with C153-RhoB-MLV particles at 1 h and 4 h post-injury. No specific fluorescent signals were observed due to C153-RhoB-MLV. Scale bar = 50 μm.
